# Can erythema multiforme be an immune sequela of IgM nephropathy? A case report

**DOI:** 10.1186/s13052-022-01373-9

**Published:** 2022-10-17

**Authors:** Francesco Messina, Laura Fagotto, Francesca Caroppo, Roberto Salmaso, Anna Belloni Fortina

**Affiliations:** 1 Pediatric Dermatology Unit, Department of Medicine DIMED, University of Padova, Padova, Italy; 2 Surgical Pathology and Cytopathology Unit, Department of Medicine DIMED, University of Padova, Padova, Italy

**Keywords:** Pediatric erythema multiforme, IgM nephropathy, Erythema multiforme

## Abstract

A 13-year-old Chinese girl attended to our Pediatric Dermatology Unit for the appearance of itchy targetoid lesions on the trunk, face and upper limbs. A skin biopsy showed histological findings typical of erythema multiforme minor. A month earlier she was admitted for the onset of a nephrotic syndrome and the renal biopsy showed an IgM nephropathy with a diffuse mesangial cell proliferation. There was no medical history of recent infections, fever, muscle or joint pain, drugs intake related to erythema multiforme and viral serology were negative.

The role of antibodies in erythema multiforme could be more relevant than suspected and the severity of erythema multiforme was reported to be proportional to the antibody-mediated complement-dependent cytotoxicity, supporting the potential pathogenetic role for humoral immunity in this subtype of erythema multiforme.

We reported the first association of erythema multiforme and IgM nephropathy in a pediatric patient providing an additional hint that an antibody-mediated process, rather than T-cell cytotoxicity, might represent the main pathogenetic mechanism in certain subtypes of erythema multiforme.

## Main text

We describe the case of a 13-year-old Chinese girl who presented to our Pediatric Dermatology Unit for the appearance of itchy targetoid lesions on the trunk, face and upper limbs.

She reported that the lesions had appeared on the hands, palms and wrists about 10 days before and then rapidly evolved affecting the trunk, upper limbs and face. She complained of intense widespread itching.

She did not report any recent infections, fever, muscle or joint pain in the last few weeks.

There was no personal or familial history of allergy to drugs or other known substances.

The girl was admitted to the Pediatric Nephrology Unit a month earlier for the onset of a nephrotic syndrome. A renal biopsy with direct immunofluorescence revealed an IgM nephropathy with a diffuse mesangial cell proliferation.

**Fig. 1:**
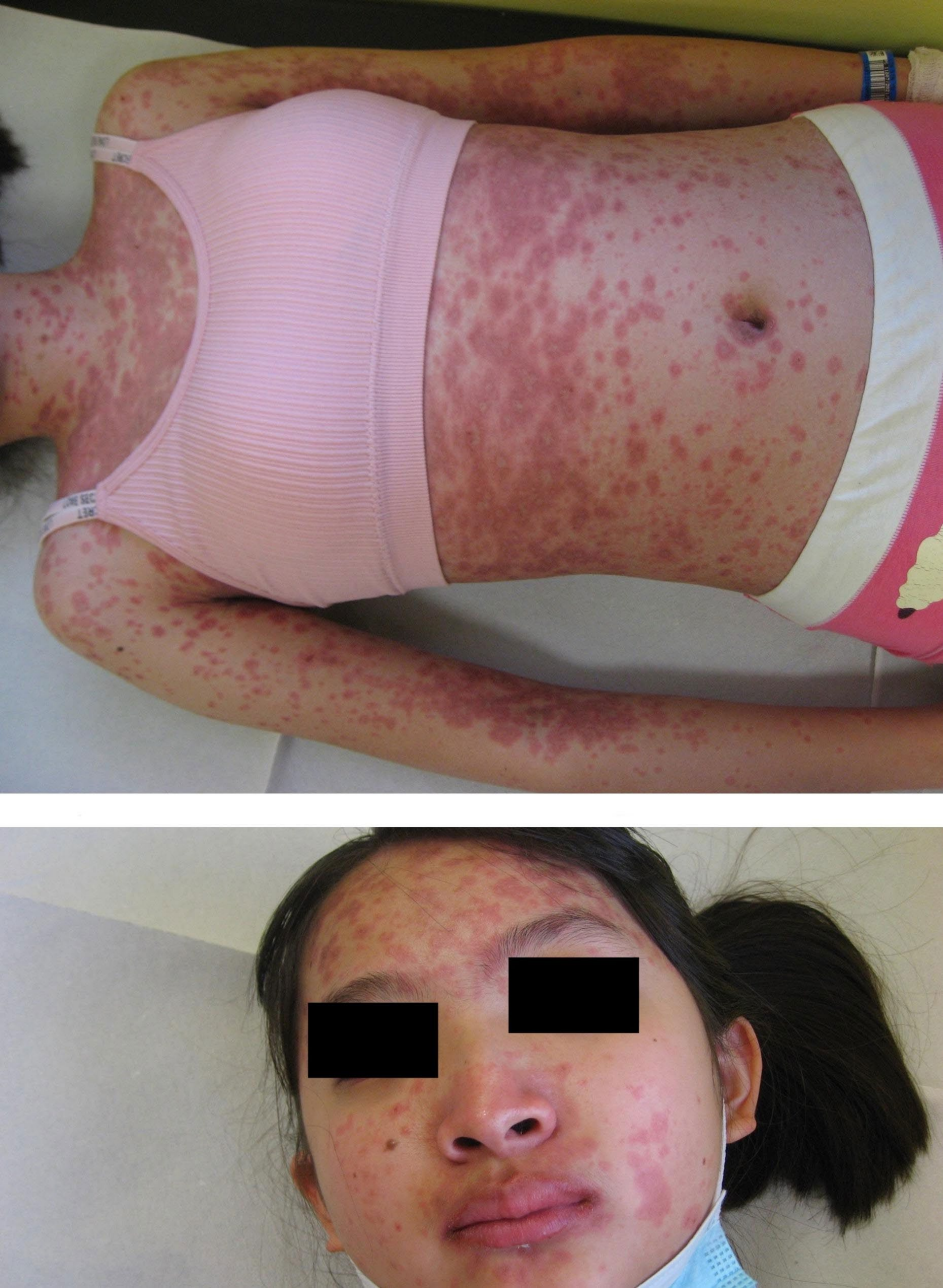
Red macules, papules, vesicles, erosions and crusts with a typical targetoid appearance, symmetrically involving the anterior region of the trunk, trunk, upper limbs and face

**Fig. 2:**
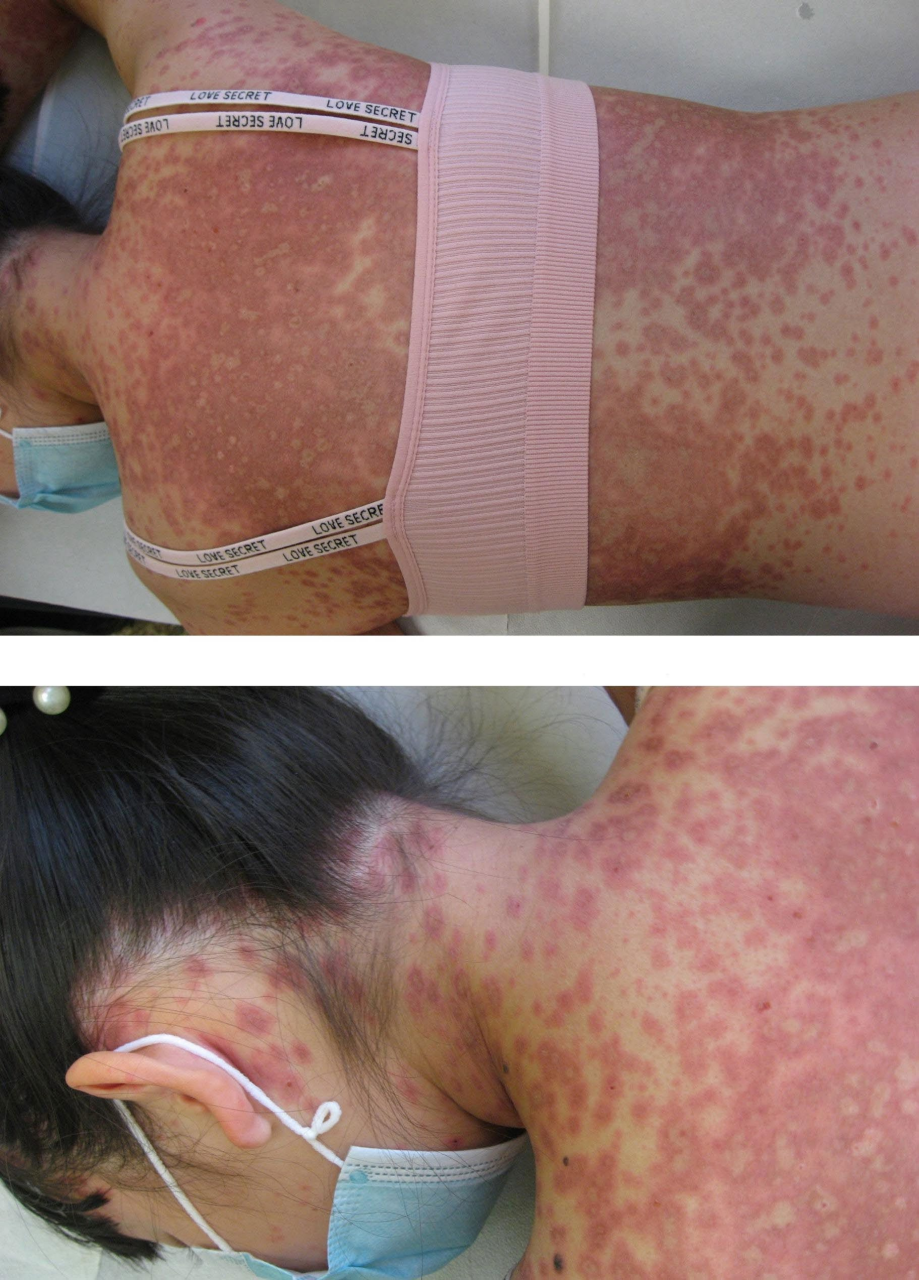
Skin targetoid lesions involving neck and posterior region of the trunk

Physical examination of the skin showed confluent red macules, papules, vesicles, erosions and crusts. Lesions had a targetoid appearance and were symmetrically localized on the trunk, upper limbs, face, inguinal region and extremities and showed a positive Nicolsky sign. Crusts were present on the sides of the mouth, with no involvement of the oral mucosa. Signs of bulbar conjunctivitis were observed. [Figures 1 and 2]

Vaginal hyperemia with mild superficial ulceration was also noted on the genitals.

Vital signs and the rest of the physical examination were normal. The complete blood count, C-reactive protein and biochemical profile were normal. Serology showed a positive ANA title (1: 320) while anti-cardiolipin, anti-beta2 glycoprotein, LAC, C3 and C4 were negative or within normal range. Cytomegalovirus serology was positive for IgG. Serology for Sars-CoV2, Epstein-Barr Virus (EBV), Parvovirus B19, Adenovirus, Enterovirus, Herpes Simplex Virus (HSV), Varicella Zoster Virus (VZV), Hepatitis B Virus (HBV), Hepatitis C Virus (HCV), Human Immunodeficiency Virus (HIV), Mycoplasma and Parechovirus have been performed twice and resulted negative.

**Fig. 3:**
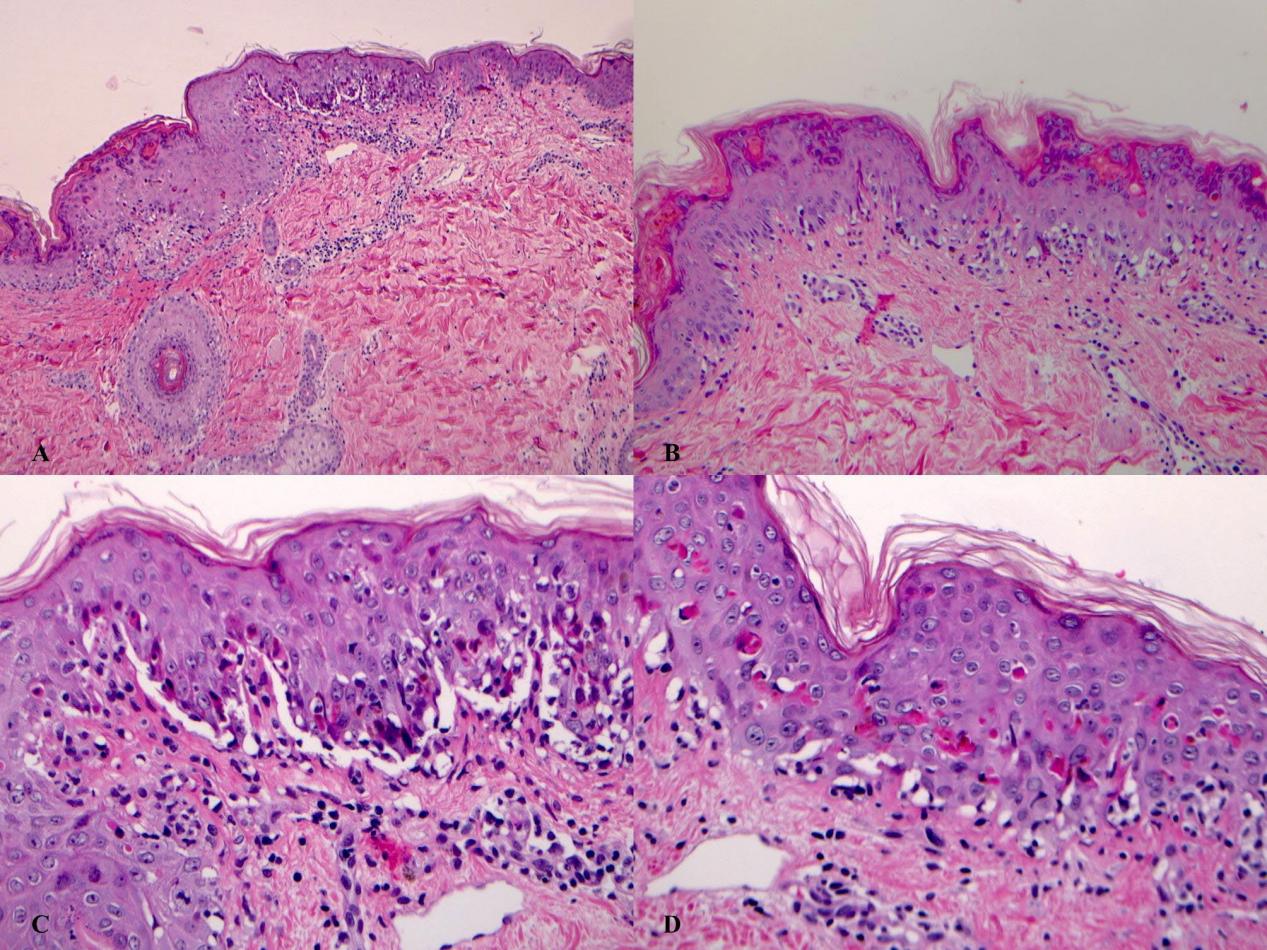
Histological sections (stained with hematoxylinand eosin – H&E) were examined by lightmicroscopy under x 10 (B), x 20 (C), x 40 (D) magnifications. Histological examination showed the typical features of erythema multiforme: hyperkeratosis, attenuation of the ridges network, necrosis and vacuolar degeneration of keratinocytes, blisters under the epidermis and discrete lymphocyte infiltrate in the papillary dermis

Suspecting an erythema multiforme (EM) minor, a skin biopsy was performed; histological examination showed hyperkeratosis, attenuation of the ridges network, necrosis and vacuolar degeneration of keratinocytes and discrete lymphocyte infiltrate in the papillary dermis with exocytosis. [Fig. 3]

Therefore, the diagnosis of EM minor was confirmed.

The patient was treated with oral prednisone 2 mg/kg/day. After an initial worsening of the symptoms, EM gradually resolved with desquamation with large flaps and remission of itch in 3 weeks.

EM is an immune-related dermatosis which is usually linked to infectious triggers, especially HSV or Mycoplasma pneumoniae. Other inductors of EM are drugs, neoplasms or other autoimmune conditions. 1,2.

In this case, the negative serologic tests excluded infectious triggers. Our patient had been treated with amoxicillin-clavulanic acid at the dosage of 1 g twice daily for the onset of a perirenal hematoma following her renal biopsy as a prophylactic measure for 18 days, until 8 days before the onset of EM. Considering that the half-life of amoxicillin-clavulanic acid is 1 h, it is reasonable to exclude that, at the date of EM onset, the drug was still present in our patient’s tissues 3. In fact, it is generally accepted that the likeliness of a drug-induced EM is related to the presence of the causative drug in the body of the patient. 4.

Therefore, considering both the presence of an alternative explanation and the time intercurred between drug exposure and the event, the association was categorized as “unlikely” using the WHO-UMC system for standardized case causality assessment. 5.

In order to exclude a drug-induced EM with a higher degree of certainty, we also performed the ALDEN score, which is validated for Stevens-Johnson syndrome and toxic epidermal necrolysis 6, which are two related conditions. ALDEN score was 1, supporting our hypothesis that amoxicillin-clavulanic acid was not involved in the onset of EM.

Our patient was diagnosed with IgM nephropathy one month prior the onset of EM. This rare renal disorder is an idiopathic disease which is connected with IgM deposition in the mesangium, and high titers of IgM or IgM immune complexes have been found in patients’ serum. 7.

IgM and C3 accumulation at dermal level is also a common feature of EM and for decades immune complex deposition was thought to be pivotal for EM pathogenesis 1, although later studies revealed that EM is a T cell-mediated disorder with IFNγ and TNFα playing a key role. 2.

However, the role of antibodies in EM could be more relevant than suspected. In fact, anti-epidermis antibodies have been revealed in mogamulizumab-induced EM 8. Interestingly, the severity of EM was reported to be proportional to the antibody-mediated complement-dependent cytotoxicity, supporting the potential pathogenetic role for humoral immunity in this subtype of EM 8. The observation that rituximab-mediated B cell depletion seems efficacious in treating some forms of EM also supports our hypothesis. 8,9.

In conclusion, we reported a case of a 13-year-old female child with an association of EM and IgM nephropathy. Our observation provides an additional hint that, in certain subtypes of EM, an antibody-mediated process, rather than T-cell cytotoxicity, might represent the main pathogenetic mechanism.

## Data Availability

not applicable.

## List of abbreviations

EBV: Epstein-Barr Virus.
HSV: Herpes Simplex Virus.
VZV: Varicella Zoster Virus.
HBV: Hepatitis B Virus.
HCV: Hepatitis C Virus.
HIV: Human Immunodeficiency Virus.
EM: Erythema multiforme.
